# Exploitation of *Neisseria meningitidis* Group B OMV Vaccines Against *N. gonorrhoeae* to Inform the Development and Deployment of Effective Gonorrhea Vaccines

**DOI:** 10.3389/fimmu.2019.00683

**Published:** 2019-04-09

**Authors:** Helen Petousis-Harris, Fiona J. Radcliff

**Affiliations:** ^1^Department of General Practice and Primary Health Care, University of Auckland, Auckland, New Zealand; ^2^Department of Molecular Medicine and Pathology, University of Auckland, Auckland, New Zealand

**Keywords:** gonorrhea, OMV vaccine, MeNZB, *Neisseria meningitides*, *Neisseria gonorrhoeae*

## Abstract

Have potential clues to an effective gonorrhea vaccine been lurking in international disease surveillance data for decades? While no clinically effective vaccines against gonorrhea have been developed we present direct and indirect evidence that a vaccine is not only possible, but may already exist. Experience from Cuba, New Zealand, and Canada suggest that vaccines containing Group B *Neisseria meningitides* outer membrane vesicles (OMV) developed to control type-specific meningococcal disease may also prevent a significant proportion of gonorrhea. The mechanisms for this phenomenon have not yet been elucidated but we present some strategies for unraveling potential cross protective antigens and effector immune responses by exploiting stored sera from clinical trials and individuals primed with a meningococcal group B OMV vaccine (MeNZB). Elucidating these will contribute to the ongoing development of high efficacy vaccine options for gonorrhea. While the vaccine used in New Zealand, where the strongest empirical evidence has been gathered, is no longer available, the OMV has been included in the multi component recombinant meningococcal vaccine 4CMenB (Bexsero) which is now licensed and used in numerous countries. Several lines of evidence suggest it has the potential to affect gonorrhea prevalence. A vaccine to control gonorrhea does not need to be perfect and modeling supports that even a moderately efficacious vaccine could make a significant impact in disease prevalence. How might we use an off the shelf vaccine to reduce the burden of gonorrhea? What are some of the potential societal barriers in a world where vaccine hesitancy is growing? We summarize the evidence and consider some of the remaining questions.

## Lessons From *N. meningitidis*

Unlike *Neisseria meningitidis* serogroups A, C, W, and Y, for which effective polysaccharide-protein conjugate vaccines have been developed, serogroup B has required alternative strategies. This is because of the poor immunogenicity of the group B capsular polysaccharide and its likely homology to fetal neural tissue (1). The original solution to this problem was the development of group B strain specific vaccines based on the outer membrane vesicle (OMV). These vaccines are based on the immunodominant protein Porin A (PorA), which is highly variable across strains, therefore their use has traditionally been considered restricted to situations where disease is dominated by a single PorA strain (2, 3). Reports on the duration of protection afforded by OMV vaccines against meningococcal disease vary according to age at vaccination and the target population, but serum bactericidal activity (SBA), which is the correlate of protection, has typically diminished among a significant proportion of vaccinees by 2 years, due to the waning of serum antibody (4).

While devastating, meningococcal disease is rare and meningococcal vaccines rely on immunogenicity data as a proxy for likely efficacy (5). This is because a randomized efficacy trial powered to detect meningococcal disease cases as a primary outcome would need in the order of 100,000 participants (6–8), rendering this approach unaffordable and impractical. Fortunately the presence of SBA provides a correlate of protection that can be used to estimate efficacy (5, 9, 10).

Estimating both the efficacy and effectiveness of meningococcal vaccines directly is hindered by the low case numbers. Both efficacy and effectiveness are generally estimated by calculating the risk of disease among vaccinated and unvaccinated persons and determining the percentage reduction in risk among each group relative to each other. Because of the low case numbers, and in the case of polysaccharide-protein-based, the significant impact on carriage (11–13) associated with meningococcal vaccines the estimates have wide confidence intervals (14).

Ultimately the public health value of these vaccines is revealed by real world experience and ecological observations on overall incidence and prevalence of disease which indirectly supports the vaccine impact. Where meningococcal vaccines are concerned estimates of 70 or 80 percent efficacy may translate to much higher effectiveness and near elimination of disease (12, 15–17).

Because the immunogenicity, efficacy, and effectiveness of OMV vaccines has generally been considered limited in terms of strain and type coverage compared with conjugate vaccines, and they have not been as widely used with less data published compared with their purer conjugated relations. While immunogenicity of OMV vaccines generally predicts efficacy (particularly for younger age groups) it may be less predictive of effectiveness (5). Less explored too are the minor components present in the OMV and their potential role in inducing not only protection against homologous PorA types but heterologous protection against a range of *Neisseria*. The traditional IgG activated complement mechanism that has perhaps driven and dominated the meningococcal vaccine field may miss a cocktail of novel antigens with powerful adjuvant effects (18).

## Potential Impact of a Gonococcal Vaccine

An effective vaccine does not have to be a highly efficacious vaccine. When the basic reproduction number of an infection is low, and a vaccine affects transmission, then disease control can be achieved with a vaccine that has moderate efficacy. While vaccines are given to individuals to protect them against disease, many vaccines also reduce transmission, thereby protecting the broader community (19). Consideration of a vaccine's effect on carriage and transmission is a vital component of immunization programme planning.

The basic reproduction number (R_0_) describes the maximal potential for spread of an infection within a population. It depends on the contact rate, the duration of the infectious period and the probability that the contact between an infectious person and a susceptible person leads to an infection (19). For the most infectious diseases (measles and pertussis) a single infectious individual entering a community of non-immune individuals can infect a further (R_0_) 12–18 and 5–17 people, respectively (20). Effective vaccines against these diseases need to be highly efficacious and affect transmission in order to successfully control or eliminate disease. They also need to provide sustained immunity, or be given as regular boosters, and be administered to 92–94% of the population (19).

In contrast, mumps has an R_0_ of around 4–7 and influenza 1.4–4. The proportion of the community that needs to be immune to prevent transmission of these diseases is 75–85 and 30–75%, respectively (20). Estimates of R_0_ for gonorrhea are in the order of 1.18–3.6 depending on the method used. The lower estimates rely on the assumption of acquired immunity after infection, the higher estimates do not assume complete acquired immunity and are therefore likely to be more accurate (21).

Using an individual-based, epidemiological simulation model the potential impact of a hypothetical gonorrhea vaccine was modeled and the prevalence of gonorrhea in a heterosexual population estimated using various assumptions of efficacy and duration of protection. The individual-based approach applies the dynamics of the *probability* that an individual is in a certain state (susceptible, infected, recovered) as opposed to a static network where an individual is in one of the states (22). In this study it was assumed that there was no immunity following resolution of natural infection. The modeling demonstrated that a vaccine of moderate efficacy could have a significant impact on the prevalence of gonorrhea if strategically implemented (23). While encouraging it does, of course, depend on the availability of a vaccine.

## From Ecological Data to Evidence

**Figure 1:**
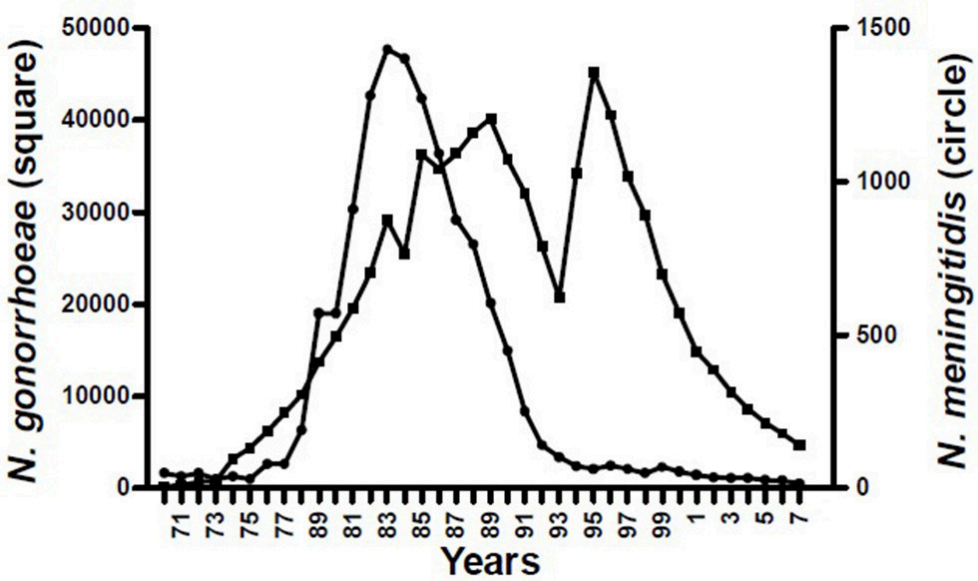
Morbidity of *Neisseria* pathogenic species in Cuba 1970–2007 (8).

**Figure 2:**
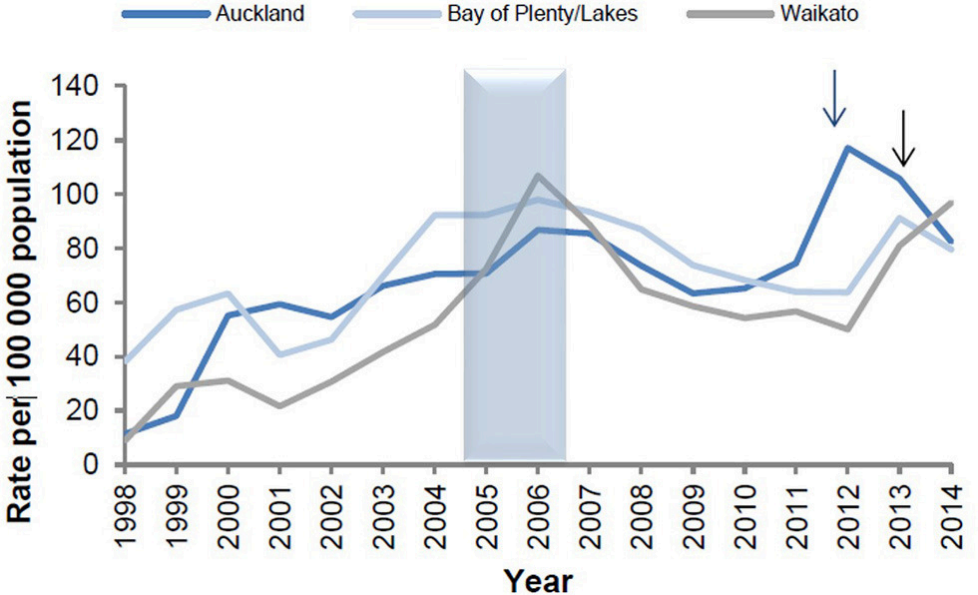
Reported gonorrhea rates in New Zealand 1998–2014, selected sentinel regions (26). Arrows denote shifts from culture testing to NAAT testing.

The epidemiological evidence from Cuba, Brazil, and New Zealand demonstrates that *N. meningitidis* OMV vaccines are possibly able to provide some broader protection against meningococcal disease (17, 24), particularly in older children and adults (25). These observations led to the hypothesis that they may affect a more distantly related bacterium. Examination of surveillance data clearly show a marked decline in the incidence of gonorrhea in Cuba following implementation of the VA-MENGOCBC (Figure 1). The pattern of decline in incidence for gonorrhea contrasts with syphilis and genital warts for which incidences have remained the same (8). This phenomenon was also observed in NZ, where a decline in reported gonorrhea cases during and shortly after use of the tailor made meningococcal Group B OMV vaccine MeNZB is evident (Figure 2). Like Cuba, no other sexually transmitted infections (STIs) described in the national surveillance reports declined during that period (26). While purely ecological, these observations suggested that *N. meningitidis* OMV vaccines might offer cross protection against gonorrhea.

This hypothesis was first tested in New Zealand where both the MeNZB exposure and gonorrhea outcome data could be linked in a retrospective case-control study. The demographic details and vaccine status of 14,730 deidentified sexual health clinic patients aged 15–30 years, who had been eligible to receive the MeNZB vaccine, were determined via the linkage to the country's National Immunization Register and database of demographic information. The outcomes of interest were laboratory confirmed gonorrhea and, as a control, chlamydia. The odds of disease outcomes in vaccinated and unvaccinated patients were compared. Individuals who had received the MeNZB vaccine were significantly less likely to be gonorrhea cases than chlamydia controls, with an adjusted OR 0.69 (95% CI 0.61–0.79%); *p* < 0·0001. This translated to a vaccine effectiveness estimate of 31% (95% CI 21–39) (27).

Following on from the case-control study a national cohort study of gonorrhea hospitalizations was conducted. The eligible cohort consisted of 1,143,897 individuals born 1984–1999 residing in New Zealand during 2004–2008 and therefore eligible to receive the MeNZB vaccine during that time. In this study administrative datasets of demographics, customs, hospitalization, education, income tax, and immunization, were linked using a national data collection called the National Integrated Data Infrastructure. The primary outcome was hospitalization with a primary diagnosis of gonorrhea. Using Cox's proportional hazards models with a Firth correction for rare outcomes, estimates of hazard ratios were generated. Vaccine effectiveness estimates were calculated as 1-Hazard Ratio expressed as percent. After adjustment for gender, ethnicity and deprivation, MeNZB vaccine effectiveness against gonorrhea associated hospitalization was estimated to be 24% (95% CI 1–42%) for the whole eligible cohort and 47% (95% CI 18–66) for those vaccinated in adolescence and therefore most likely to be at risk for gonorrhea during the follow-up period (28). While limited by small numbers the findings supported the earlier case-control study.

The MeNZB vaccine was developed to control a meningococcal group B epidemic and it is no longer available. However, the same OMV used in the MeNZB vaccine is included in the new generation meningococcal group B 4CMenB (Bexsero) vaccine. Bexsero also includes three recombinant proteins that are conserved across *N. meningitides* [Neisserial Heparin Binding Antigen (NHBA), factor H binding protein (fHbp), and Neisseria Adhesion A (NadA)], two of which are shared variably with Neisseria *gonorrhoeae (N. gonorrhoeae)* (29). NHBA and fHbp, along with accessory fusion proteins GNA1030 and GNA2091 in the formulation, are capable of inducing immune responses against the gonococcus (30, 31). If additional immunogenicity and cross protection is afforded by this new generation vaccine, on account of the additional antigens and synergistic combination, then the potential for effectiveness is likely to be higher than that demonstrated by the MeNZB vaccine.

A mass vaccination campaign in Quebec, Canada, using Bexsero, provided an opportunity to observe a potential vaccine effect on gonorrhea. In 2014 Bexsero was administered to individuals aged 6-months to 20-years of age. Gonorrhea and chlamydia notifications to public health authorities during the pre-vaccination period and post-vaccination period (2006–June 2017) were analyzed and the impact of the campaign estimated by a Poisson regression model. Vaccine coverage was 82% in the target group. There were 231 gonorrhea cases reported among persons 14 years and older (IR: 8.4/100,000 person-years) in the region of the mass campaign during the study period. A decrease in the number of cases and risk among individuals 14–20 years was observed during the post-vaccination period. In contrast, it increased in those 21 years and older. As observed in both New Zealand and Cuba, chlamydia infections increased among both the vaccinated and unvaccinated age groups. The estimate of vaccination impact was a risk reduction in gonorrhea of 59% (95% CI: −22 to 84%; *P* = 0.1) (32). While the confidence intervals are wide and include “0” this is likely due to the low study power.

Together the NZ, Cuban, and Canadian data suggest that we likely already have a vaccine to hand, that if used strategically, could impact on the prevalence of gonorrhea. But what are the mechanisms?

## Strategies to Define the Mechanisms of Cross-Protection

The intriguing observation that vaccination with meningococcal B OMVs confers a degree of protection to gonorrhea raises some interesting questions about possible mechanisms of action. Historically there have been some major confounders for development of a gonococcal vaccine—earlier trials proved unsuccessful, there are no defined correlates of protective immunity and the optimum route of delivery is unknown. The MeNZB data suggest it may be possible to elicit protective immunity to gonococci with a parenteral vaccine and there is now an opportunity to examine resources generated both from the clinical trials of this vaccine and New Zealand's large MeNZB primed cohort for clues as to how this vaccine confers this effect.

The lack of known correlates of protective immunity is a major stumbling block to identifying vaccine candidate antigens or novel approaches for developing a gonococcal vaccine. Population data show that prior exposure does not protect individuals from re-infection (33) and human challenge studies have demonstrated that recently infected individuals remain susceptible to re-infection with the challenge strain (34). Longitudinal studies of individuals regularly infected with gonorrhea have proved invaluable in demonstrating how host antibody mediated responses to selected antigens have been used to subvert the immune response. Gonococci can be typed into serovars on the basis of expression of variants of a major outer membrane protein, porin (PorB). Both *porB* typing alone or in combination with additional molecular analyses have shown that *por* mutates in response to immunological pressure, suggesting that the effective immunity to gonococci may develop, but is confounded via a shifting of phenotype by the bacteria (35, 36). Development of an elevated antibody response to another major outer membrane protein, reduction modifiable protein (Rmp)/protein III, is associated with enhanced susceptibility to infection (37). *In vitro* studies have shown that IgG antibodies to Rmp prevent other potentially protective antibodies from initiating bactericidal activity to gonococci (38). However, individuals with high occupational exposure to gonococci do eventually develop serovar-specific immunity (35), suggesting a vaccine may ultimately be feasible (39). Experimental, self-limiting infection of human subjects with gonococci results in development of modest local and systemic specific antibody responses, with a suggestion that serum antibody responses to lipo-oligosaccharides (LOS) may confer a degree of protection from re-infection (34).

Several prospective gonococcal vaccines have been tested in humans without success. To date these attempts have included parenteral administration of non-adjuvanted material including partially lysed whole bacteria (40), pilin (41), and protein I/PorB, a major porin/outer membrane protein [reviewed in (42)]. One of the most promising pre-clinical vaccine candidates identified to date is a highly conserved oligosaccharide epitope (2C7) common to most gonococcal isolates—targeting this epitope elicits sialylation-independent bactericidal activity *in vitro* and leads to enhanced clearance of infection in mice (43, 44). Pre-clinical evaluations suggest that gonococcal OMVs are also a promising vaccine candidate (45–47) but they have not been tested in humans. In contrast serogroup B *N. meningitidis* was the first organism to be screened for meningococcal vaccine candidate antigens using a sophisticated genomic-led approach termed “reverse vaccinology,” which led to the selection of the highly conserved vaccine candidate antigens incorporated into Bexsero (48). Importantly though for full efficacy and enhancement of coverage to a broader range of subserotypes and better immunogenicity in younger age groups, this vaccine still requires the addition of OMV from MeNZB, with porin A considered to be a particularly important constituent (49, 50).

The OMVs are comprised of a complex mix of periplasmic, cytoplasmic, and outer membrane proteins (51). The dominant components include outer membrane proteins such as porin A, porin B, Rmp, NspA, and the OpcA invasin which are incorporated into vesicles in conjunction with lipopolysaccharide [summarized in (52)]. An immuno-proteomics analysis of the related Cuban meningococcal OMV vaccine suggests that the antibody response is primarily targeted to these major antigens (53). Population genomics has demonstrated the close genetic relationship between *N. meningitidis* and *N. gonorrhoeae* (50) suggesting OMVs are likely to be the source of numerous conserved vaccine candidates, both protein and glycolipid. Screening of gonococcal OMVs from multiple strains with human anti-meningococcal sera would be an effective approach to identify cross-reactive, conserved “human relevant” antigens. Although this approach is likely to reveal multiple cross-reactive antigens, some of which are unlikely to be suitable vaccine candidates, selection of novel candidate antigens can be informed and complemented by the rapid expansion of knowledge of gonococcal biology. For example, conserved novel antigens of importance for survival of the gonococcus have recently been identified by quantitative proteomics, which has successfully been applied to cell envelope and OMVs from multiple strains of *N. gonorrhoeae* (54).

As *N. gonorrhoeae* is an exclusive human pathogen the relevance of animal models, particularly non-primate models, is contentious. Female mice can be transiently infected with *N. gonorrhoeae* if they are pre-treated with estradiol and inoculated during the pro-estrous phase (55), but lack important features such as carcinoembryogenic antigen-related cell adhesion molecules (CEACAM; CD66) on neutrophils and epithelial cells, which are typically targeted by the gonococcal opacity associated proteins (Opas) (56, 57). Gonorrhea infection in mice does recapitulate several known features of human infection, eliciting a polarized neutrophilic Th17 driven response and production of pro-inflammatory cytokines (58–60). Conversely, accelerated clearance of the infection is linked to a Th-1 driven immune response in mice, particularly the production of interferon-γ, in conjunction with the development of both systemic and local IgG and A (45, 61–63). Of note, vaginal vaccination with gonococcal OMVs delivered with encapsulated IL-12 can elicit enduring protection to multiple distinct gonococcal isolates (45) and intranasal administration of gonococcal OMVs can expedite resolution of infection (46), however it is not yet known whether parenteral vaccination with either gonococcal or meningococcal OMVs can elicit a comparable effect.

Although gonococci can infect other mucosal sites, the most important site of entry is the genital mucosa. Unlike other mucosal locations, the genitals lack inductive sites for local antibody production and IgG (produced locally and systemically), not IgA, is considered to be the most important class of antibody (64). It has been traditionally accepted that the optimal way of developing vaccine-elicited immunity to mucosal pathogens is to immunize by the mucosal route, preferably one which mimics the natural route of infection (65). Accordingly there are several examples of highly effective vaccines—most notably the live oral vaccine for poliomyelitis—which are administered orally, although further refinements in this arena have been stymied by the lack of safe, effective mucosal adjuvants. More recently it has been proposed that parenteral vaccines may be an equally feasible means of stimulating strong and appropriate protective mucosal responses (66). Of particular relevance to gonorrhea, the Human Papillomavirus (HPV) vaccine is comprised of an adjuvanted preparation of virus-like particles given intramuscularly, which results in the development of enduring antibody responses in both systemic (serum) and local (cervicovaginal) sites, with a strong correlation between IgG levels in the two locations, supporting the possibility of serum transudation or exudation into the genital mucosa (67). Evidence from gonococcal vaccine studies in mice also indicate that antibody responses—including systemic IgG responses—are associated with protective immunity to gonococci, suggesting serum antibody responses can reasonably be used as a correlate of protection (45).

But how does MeNZB, which is reported to confer a relatively short-lived period of protective immunity in infants and toddlers (4, 68), confer cross-protective immunity to gonococci for some years after vaccination? An elevated SBA is the benchmark for verifying sustained protective immunity to group B meningococci, but there may well be other aspects of the vaccine initiated immune responses that are equally or more important for development of immunity to gonococci. Specific mucosal immune responses are likely to be necessary for limiting the development—and ideally transmission—of symptomatic gonorrhea infections. While the systemic antibody responses elicited by MeNZB have been thoroughly examined, there have been no long term studies on the development of the mucosal antibodies in response to vaccination. An assessment of adults shortly after vaccination showed either no change or very modest increases in salivary antibody responses in response to parenteral vaccination with the Norwegian MenBVac (69) or MeNZB (70). Conversely, anti-meningococcal salivary IgA responses were reported to increase with age and/or meningococcal carriage (71), suggesting that an examination of the long-term effects of vaccination in childhood may yield useful information on whether nasopharyngeal acquisition and/or carriage of commensal *Neisseria* can boost mucosal antibody responses. Given the issue of rising rates of oral gonorrhea and evidence of oral-genital transmission of infection (41), induction of strong mucosal immune responses in both sites is likely to be important.

Analysis of specific cellular responses in the tonsils suggest that parenteral vaccination of adults with MeNZB results in re-programming of the mucosal immune response to meningococci in the nasopharynx (70). OMVs contain a complex package of virulence factors, TLR agonists, and other secreted or membrane associated components that interact and modulate host immunity (72). Detergent extracted MeNZB OMVs are reported to consistently contain ~100 distinct proteins predominantly from the outer membrane compartment (73) and although delivered with an adjuvant, they are also intrinsically immunogenic. Perhaps OMVs from mucosal pathogens such as *Neisseria* have the capacity to stimulate homing and development of immune effector cells at mucosal sites after parenteral immunization? This may be important for eliciting strong mucosal immune responses (66) as has recently been reported for the detoxified form of heat-labile toxin (dmLT) (74) and linked to properties of the adjuvant itself, rather than the route of administration. This would be an interesting concept to explore further in mice vaccinated parenterally or mucosally with OMVs; or translated to humans through application of emerging technologies in the immunogenomics and systems biology arena (75) with the potential to provide vital information to support the utility of this class of vaccines.

Human mucosa, particularly the oropharynx, is frequently host to several different species of *Neisseria*. Genetic analyses of both pathogenic and non-pathogenic *Neisseria* show they are closely related, with evolutionary studies suggesting frequent exchange of genetic material including virulence genes (76). Notably, early exposure to commensals such as *N. lactamica* may not only confer some degree of protection from *N. meningitidis*, but could be an approach for identifying novel vaccine candidate antigens (77). Carriage of non-pathogenic *Neisseria* may also enhance the development of immunity to *Neisseria* OMV vaccines (78). *N. meningitidis* and *N. gonorrhoeae* are most closely related and primarily separated from the remaining members of the genus by the presence of additional virulence genes (76). The impact of vaccination with meningococcal OMVs, which contain multiple highly conserved antigens common to many *Neisseria* species, on nasopharyngeal colonization has not yet been determined. A genetic analysis of the additional antigens in Bexsero suggest that this vaccine could impact non-target *Neisseria* species as both NHBA and the additional fusion proteins GNA1030 and GNA2091 contained in Bexsero are highly conserved across both pathogenic and commensal *Neisseria*, whereas *N. gonorrhoeae* does not express NadA and this bacterium contains only one of three possible variants of fHbp (79, 80). It has been suggested that NHBA could form the basis of a putative gonococcal vaccine, but would likely require augmentation with additional conserved proteins to enhance the effectiveness of such a vaccine (80). MeNZB anti-sera is highly likely to be a useful source for identifying additional highly conserved gonococcal immunogens. The utility of this approach has been demonstrated in rabbits, which develop cross-reactive antibodies to several strains of gonococci by ELISA and western blot in response to vaccination with MeNZB/Bexsero OMVs (31). Human MeNZB anti-sera will almost certainly show a similar level of cross-reactivity, with the potential complication of reactivity to antigens common to commensal *Neisseria* species.

New Zealand currently has a large cohort of individuals at or approaching the age of prime interest for studying the long-term impact of the MeNZB vaccine. Notably there is an opportunity to examine recall responses ~10 years post-priming in a population of adolescents and/or young adults, which is a good match for the likely timing of administration of a gonococcal vaccine and corresponds with the age groups at greatest risk of contracting gonorrhea. The impact of boosting these individuals with Bexsero (as the source of MeNZB OMVs) on both local and systemic antibody responses as well as the development of cellular immunity has the potential to provide valuable data on the possible targets or mechanisms of cross-reactive immunity. A large collection of sera also remains from the original MeNZB clinical trials, which were commenced in adults (81), followed by pre-teens (82), toddlers then infants (83) with serum samples obtained prior to and at regular intervals during vaccination. Untouched duplicate samples were retained after the completion of these trials and can be accessed for investigation of anti-gonococcal responses. Meningococcal SBA titres were quantified as part of the MeNZB vaccine development programme, which offers a unique opportunity to not only determine whether MeNZB vaccination elicited a bactericidal antibody response to gonococci in humans, but also to ascertain whether this correlates with elevated anti-meningococcal SBA titres.

A key question remains as to which approaches would be the most appropriate for examining possible cross-reactive immune responses to gonococci in these vaccinated individuals. Induction of functional antibody responses such as complement-mediated bacterial killing, inhibition of binding to reproductive tract epithelial cells and stimulation of opsonophagocytosis are generally considered to be suitable starting points. The development of an increased serum bactericidal antibody (SBA) response is the standard for determining the protective efficacy of vaccines to meningococci (84–86) whilst the presence of bactericidal antibodies to gonococci are frequently used as a likely surrogate of protective immunity in pre-clinical studies (47, 87, 88). Cross-reactive bactericidal antibodies are, unsurprisingly, likely to be directed to gonococcal lipopolysaccharides and surface proteins (89). Therefore, analyzing SBA responses is a valid starting point, although it will be important to incorporate multiple gonococcal strains to confirm broad, cross-protective bactericidal activity to gonococci. A limitation of the SBA is that it requires use of bacteria that are resistant to complement-mediated killing by normal serum, whereas serum sensitivity is reported to be common in gonococci after *in vitro* culture (90). Serum resistance can be induced by the use of additives to sialylate LOS (91, 92) and screening of resistant phenotypes is more likely to represent the low susceptibility to serum-mediated killing seen *in vivo*. Enhanced opsonophagocytosis is considered to be another likely correlate of protective immunity to gonococci and this can readily be inferred by detection of C3b deposition on the surface of the gonococci using flow cytometry (93), as a pre-cursor to MAC-mediated lysis, and corroborated by determining whether opsonophagocytic uptake of gonococci by a neutrophil-like cell line, such as retinoic acid differentiated HL-60 cells (94), is enhanced in the presence of immune sera.

A critical step in the pathogenesis of *N. gonorrhoeae* is adherence to target epithelial cells where initial adherence is predominantly mediated by pili, followed by tight attachment to CEACAM via expression of OpA. The interactions of gonococci with human reproductive tract cell lines (95, 96) and primary cultures (97) have been described and these model systems can be used to establish whether introduction of sera from MeNZB vaccinated individuals inhibits adherence or invasion of gonococci. These sera can also be used to determine whether there is a quantifiable increase in antibodies to gonococcal OMVs or cell surface exposed antigens by ELISA or development of cross-reactive antibodies to antigens conserved across multiple isolates of *N. gonorrheae* using similar immunoproteomics approaches to those applied to *N. meningitidis* (53, 98, 99). Both approaches can be used to identify or validate potential gonococcal vaccine candidates, in conjunction with pre-clinical testing in *in vivo* models.

There have been few studies on the development of cellular immune responses to MeNZB (70) or other meningococcal OMV vaccines in adults (100, 101). None have examined early immune kinetics in naïve individuals, the development of recall responses, or induction of responses to gonococcal OMVs. The type of cellular response required to prevent gonococcal infection in humans is unknown, but murine studies link development of protective immunity with Th-1 immune responses (45). An assessment of Th profiles in response to stimulation with gonococcal antigens could ascertain whether MeNZB vaccination causes a similar skewing in humans.

Mining resources from New Zealand's MeNZB vaccination programme may be fruitful for identifying potential correlates of immunity in human subjects. Ultimately a prospective clinical trial or high quality observational study with a large cohort of high risk individuals will be necessary to acquire a complete and accurate picture of how well a vaccine containing OMVs (or other potential antigens) will perform in reducing rates of gonorrhea.

## Societal and Policy Issues in Gonococcal Vaccine Deployment

Modeling has suggested that a vaccine with moderate protection might have a significant effect on the burden of gonorrhea (23). Considering Bexsero as a candidate intervention, how would we best use it?

While we do not yet know the mechanism of protection induced by these OMV vaccines that has resulted in some resistance to gonorrhea we might assume that it is not long lasting based on experience with protection against meningococcal disease as well as the waning observed in the New Zealand case-control study (27). There are still questions to be answered about the effectiveness of Bexsero. It does appear broadly protective against meningococcal disease, including against the hypervirulent Group W strain, which is increasing in prevalence in some countries (102, 103). However, to what extent this affects carriage in adolescents, and duration of protection at the population level remain to be demonstrated (103, 104). Therefore, in order to optimize protection against gonorrhea such a vaccine would need to be delivered prior to sexual debut and use of boosters possibly maintained throughout the risk period. Early adolescence also happens to coincide with a high risk period for meningococcal disease. Administering a dose of Bexsero during early adolescence in a population previously primed in infancy, or two doses in a previously naive population, might be a pragmatic strategy to reduce gonorrhea whilst at the same time improving the community immunity to meningococcal disease. If protection was demonstrated to wane during the risk period a further dose could be considered to maintain protection.

Even the most efficacious vaccine cannot prevent disease if it is not used. Funding and policy aside perhaps the greatest challenge facing immunization programmes today is the growing presence of vaccine hesitancy (105, 106). The dream of global measles and rubella elimination is unraveling as a tide of trolls, bots, and organized opposition, facilitated by social media, plays havoc with trust and confidence (107–109). Vaccine coverage rates have dived in many countries and measles resurgence is occurring among populations that had previously achieved elimination status, such as the America's and some European countries (110). How might a gonorrhea vaccine fair in this environment of growing public resistance to vaccines?

If we consider the societal factors that might be relevant to a vaccine against a disease that is largely sexually transmitted then we need look no further than the experience with the human papillomavirus (HPV) vaccines. The focus of the original marketing of HPV vaccines was prevention of cervical cancer as opposed to prevention of a sexually transmitted infection (111, 112) however, skepticism about the vaccine effectiveness and safety arose quickly from a variety of quarters (113, 114). Even with the focus on cancer there has been ongoing public outrage fed by organized lobby groups since 2007, when the first vaccine was licensed (115). After the introduction of HPV vaccine there was a shift toward a more conservative backing for the anti-vaccine movement. Presumably this was because despite the vaccine being marketed as an anti-cancer vaccine the fact that the virus is primarily sexually transmitted invoked discomfort among those with conservative and religious views about sex (116). Consequently whilst some countries have delivered to over 70–80% of the target population (117) others have fared less well with some countries either failing to implement a programme, or experiencing interruption to programs due to widespread movements aimed at discrediting the vaccine (118). Some countries have had their previously high coverage eroded to below 30% (119).

Given the multiple challenges in marketing and delivering a vaccine against a sexually transmitted infection across diverse cultures there may be an argument for desexualizing it and packaging it as a vaccine against *Neisseria*. However, efforts to desexualize vaccines can backfire. Withholding information that is seen to be less agreeable to the public can result in accusations of paternalism (116). This will likely result in the erosion of trust. How public health officials communicate the facts about a gonorrhea vaccine across multi-cultural societies will likely have an impact on acceptance.

Some societies have chosen to place HPV vaccine at the 9-year age mark, other societies have elected to place it at the 12–13 year mark. While the 9-year mark might desexualize the vaccine, the luxury of this choice is unlikely to be an option for a gonorrhea vaccine. The HPV vaccine, like the Hepatitis B vaccine, has a long duration of protection (120). It is unlikely that a gonorrhea vaccine based on current options will provide long-term protection, therefore placement in a national immunization programme will need to be at the age that provides highest immunity just before sexual debut.

While research continues into gonorrhea vaccine antigen discovery there is also a need for further data on the two OMV-containing *N. meningitidis* vaccines currently available (Bexsero and VA-MENGOCBC) that might have some utility if deployed into a sexually active population. For example, further knowledge about the effect of age and immunological experience on the vaccine response, along with boosting responses in older children, and adults who have been primed in infancy or early childhood. Other outstanding questions include the number of doses required to optimize responses to the gonococcus both qualitatively and quantitatively, and the potential to affect carriage.

Several lines of evidence suggest a vaccine that could impact on the growing burden of gonorrhea already exists. While the mechanisms are not yet understood, elucidating these will contribute to the ongoing development of high efficacy vaccine options for this disease. In order to successfully deploy a vaccine that could impact on the prevalence of gonorrhea the development of formulations that target all pathogenic *Neisseria* species might be the most socially acceptable, while also the most pragmatic when considering implementation into already crowded immunization schedules.

## Author Contributions

All authors listed have made a substantial, direct and intellectual contribution to the work, and approved it for publication.

### Conflict of Interest Statement

The authors declare that the research was conducted in the absence of any commercial or financial relationships that could be construed as a potential conflict of interest.
